# *SMC2* ablation impairs bovine embryo development shortly after blastocyst hatching

**DOI:** 10.1530/REP-24-0211

**Published:** 2024-10-03

**Authors:** Pérez-Gómez Alba, Flores-Borobia Inés, Hamze Julieta Gabriela, Galiano-Cogolludo Beatriz, Lamas-Toranzo Ismael, González-Brusi Leopoldo, Ramos-Ibeas Priscila, Bermejo-Álvarez Pablo

**Affiliations:** 1 Department of Animal Reproduction, INIA, CSIC, Madrid, Spain; 2 Department of Cell Biology and Histology, Universidad de Murcia. International Excellence Campus for Higher Education and Research (Campus Mare Nostrum), Murcia, Spain

## Abstract

**In brief:**

Bovine embryos lacking SMC2 (a core component of condensins I and II) are unable to survive maternal recognition of pregnancy. *SMC2* KO embryos are able to form blastocysts, exhibiting a reduced cell proliferation ability, and arrest their development shortly after hatching.

**Abstract:**

Condensins are large protein complexes required for chromosome assembly and segregation during mitosis and meiosis. Mouse or bovine embryos lacking SMC2 (a core component of condensins I and II) do not complete development to term, but it is unknown when they arrest their development. Herein, we have assessed the developmental ability of bovine embryos lacking *SMC2* due to a naturally occurring mutation termed HH3 (Holstein Haplotype 3) or by CRISPR-mediated gene ablation. To determine if embryos homozygous for the HH3 allele survive to maternal recognition of pregnancy, embryonic day (E)14 embryos were flushed from superovulated carrier cows inseminated with a carrier bull. Mendelian inheritance of the HH3 allele was observed at E14 conceptuses but conceptuses homozygous for HH3 failed to achieve elongation and lacked an embryonic disc. To assess the consequence of the ablation of condensins I and II at earlier developmental stages, *SMC2* KO bovine embryos were generated *in vitro* using CRISPR technology. *SMC2* KO embryos were able to form blastocysts but exhibited reduced cell proliferation as evidenced by a significantly lower number of total, trophectoderm (CDX2+), and inner cell mass (SOX2+) cells at Day (D) 8 post-fertilization compared to their WT counterparts and were unable to survive to D12 *in vitro*. *SMC2* ablation did not alter relative telomere length at D8, D12, or E14. In conclusion, condensins I and II are required for blastomere mitosis during early development, and embryos lacking those complexes arrest their development shortly after blastocyst hatching.

## Introduction

Genotyping programs pursuing the genetic improvement of cattle breeds have uncovered multiple haplotypes (alleles) involved in cattle reproductive performance, including several deleterious haplotypes which are never found in homozygosity at birth (VanRaden *et al.* 2011). Homozygous embryos for a deleterious haplotype—termed double-carriers (DC) – arrest their development at some point between conception and birth, thereby providing information on the essential role of a gene during embryonic or fetal development (Perez-Gomez *et al.* 2024*a*). Holstein Haplotype 3 (HH3) is a deleterious allele present in ~5% of Holstein Friesian cattle (VanRaden *et al.* 2011) that is composed of a non synonymous SNP (T/C) within exon 24 of the structural maintenance of chromosomes 2 (*SMC2*) gene, which substitutes amino acid 1135 from phenylalanine (TTC) to serine (TCC) within the NTPase domain of SMC2 (Daetwyler *et al.* 2014; McClure *et al.* 2014). SMC2 protein is a core subunit of condensins I and II (Stray *et al.* 2005), large protein complexes required for chromosome assembly and segregation during mitosis and meiosis (Hirano 2016), and its essential role in embryo/fetal development is conserved in mammals, as *Smc2* KO mouse embryos do not survive to term (Nishide & Hirano 2014).

The developmental stage at which mammalian embryos lacking both condensin complexes (I and II) arrest their development is currently unknown, as the developmental potential of *SMC2* KO embryos has not been precisely determined and no study has been conducted on embryos lacking the other core component of condensins I and II (SMC4). Embryo mortality in HH3 DC bovine embryos (*SMC2* KO) has been estimated to occur before the second month of gestation according to the reproductive performance data available from cattle genotyping programs (McClure *et al.* 2014). Unfortunately, such data do not allow for the identification of when HH3 double-carrier (*SMC2*-null) embryos arrest their development within the first 2 months of gestation. Prior experimentation in mice has not addressed that question, as the earliest time point analyzed was E12.5 (Nishide & Hirano 2014), a fetal stage not reached by bovine embryos by the second month of gestation (Krog *et al.* 2018; Theiler 1989), when no *Smc2* KO mouse fetuses were recovered.

From conception to the second month of gestation, the cattle embryo transits through critical developmental stages (Perez-Gomez *et al.* 2021). The initially undifferentiated blastomeres differentiate into inner cell mass and trophectoderm cells, leading to the formation of a blastocyst around embryonic day 7 (E7). Following blastocyst expansion, the embryo hatches from the zona pellucida (~E9) and the inner cell mass differentiates into the epiblast—which will form the embryo proper—and the hypoblast—which will form the extra-embryonic membranes together with the trophectoderm. After hatching, hypoblast proliferates to form an inner layer beneath the trophectoderm by E11 (Maddox-Hyttel *et al.* 2003) and both extra-embryonic membranes experience massive growth required to signal pregnancy to the uterus and prevent luteolysis by E15–16 (Forde *et al.* 2011; Northey & French 1980). Meanwhile, the epiblast develops an embryonic disc where gastrulation starts around E14–15 (van Leeuwen *et al.* 2015) and leads to the differentiation of the three germ layers (ectoderm, mesoderm, and endoderm) prior to implantation (E19–20 (Bazer *et al.* 2009)). Following implantation, several critical structures identifiable by ultrasonography – such as the spinal cord, forelimbs, hindlimbs, optic lens, and ribs – are formed before E60 (Curran *et al.* 1986). To test the hypothesis of condensins being required for mammalian preimplantation development, this study has determined which of these developmental stages can be reached by mammalian embryos lacking condensins I and II (*SMC2* KO) by conducting *in vivo* experimentation with HH3 single-carrier (SC) animals and *in vitro* experimentation with *SMC2* KO bovine embryos generated by CRISPR.

## Materials and methods

### *In vivo* conceptus collection

**Figure 1:**
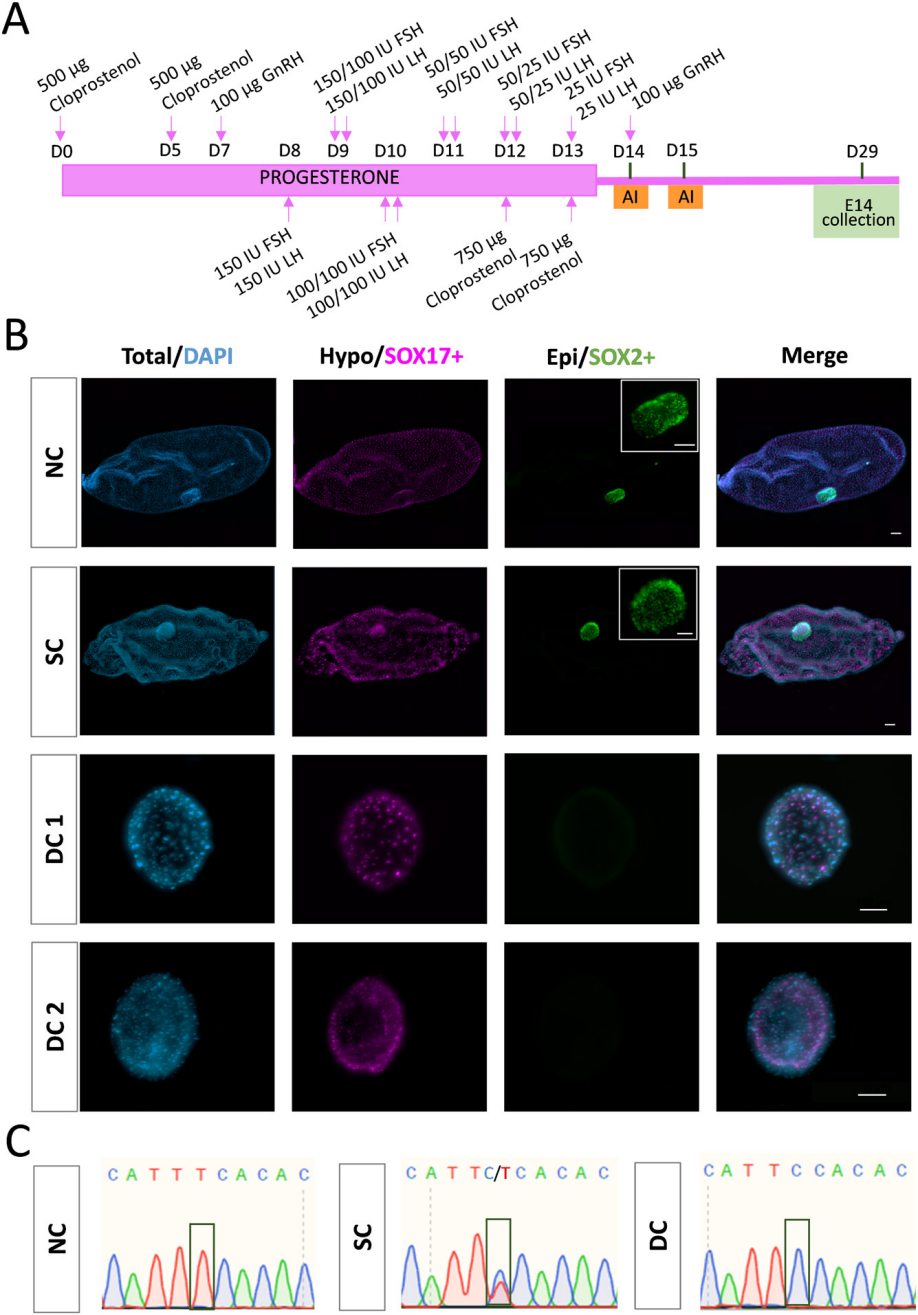
Conceptus elongation is impaired in HH3 double-carrier (DC) conceptuses. A) Superovulation protocol to obtain *in vivo* elongated conceptuses. AI, Artificial Insemination; E, Embryonic day. B) Immunohistochemistry to detect SOX2 (epiblast) and SOX17 (hypoblast); cell nuclei counterstained with DAPI. Representative pictures of tubular NC and HH3 SC conceptuses, and the two HH3 DC embryos obtained at E14. Scale bar: 100 µm for conceptuses; 50 µm for embryonic disc magnifications. C) Representative Sanger sequencing chromatograms from PCR products of conceptuses recovered from a cross of SC individuals. The image on the left corresponds to an NC conceptus (displaying TTC codon), the middle image depicts an SC conceptus (carrying both TTC and TCC –HH3- alleles), and the image on the right shows a DC conceptus (harboring the TCC codon –HH3 allele- in both chromosomes).

Cattle embryos were collected at a commercial cattle farm following conventional protocols employed for reproductive management (Ramos-Ibeas *et al.* 2020), involving a superovulation protocol detailed in (Fig. 1A) and approved by Madrid Region Authorities (PROEX 040/17). Four stimulated HH3 carrier cows were inseminated with semen from a carrier bull of proven fertility, and embryos were collected from the uterus 14.5 days after the first insemination (E14) by non-surgical flushing using a Luer catheter. Immediately after collection, embryos were fixed in 4% paraformaldehyde (PFA) for 15 min at room temperature (RT), washed, and stored in PBS supplemented with 1% bovine serum albumin (PBS-1% BSA) at 4°C until analysis.

### Generation of CRISPR components for oocyte microinjection

Capped polyadenylated Cas9 messenger RNA (mRNA) was *in vitro* transcribed by the mMESSAGE mMACHINE T7 ULTRA kit® (Life Technologies) using the plasmid pMJ920 (Addgene 42234) linearized with BbsI and treated with Antarctic phosphatase (NEB) and purified using the MEGAClear kit (Life Technologies) (Bermejo-Alvarez *et al.* 2015). Single guide RNA (sgRNA) against the 24th exon of *SMC2* (the exon containing HH3 SNP) was designed using CRISPOR (Concordet & Haeussler 2018) and synthesized and purified using the Guide-it sgRNA *In Vitro* Transcription Kit® (Takara) using a primer containing the T7 promoter and the sgRNA sequence (Table 1). Genome edition and KO generation rates were assessed by genotyping all embryos developing into blastocysts as described below.

**Table 1:** Details of primers used. The target sequence in the CRISPR system is presented in bold and underlined.

ID	Sequence (5′→3′)		Use
	Forward	Reverse	
Geno	TCAGCGCTTTGGCAAAAAGT	AGCCTCTTTCCTTCTGGAACC	To genotype E14 conceptuses by Sanger
GenoIl	TCGTCGGCAGCGTCAGATGTGTATAAGAGACAGGGTAGCAGATTTGCTTCAGCG	GTCTCGTGGGCTCGGAGATGTGTATAAGAGACAGTCTGAGGCTCTTTTTGGTTCT	To genotype D8/12 embryos, including Illumina overhangs (underlined)
Telq	CGGTTTGTTTGGGTTTGGGTTTGGGTTTGGGTTTGGGTT	GGCTTGCCTTACCCTTACCCTTACCCTTACCCTTACCCT	To analyze relative telomere length
Sat216	TGGAAGCAAAGAACCCCGCT	TCGTGAGAAACCGCACACTG	To analyze relative telomere length
T7 Guide-it	CCTCTAATACGACTCACTATA **GGATGAGGTCGATGCAGCCC** GTTTAAGAGCTATGC		To produce gRNA against *SMC2*, target sequence in bold and underlined.

### *In vitro* production of *SMC2* KO embryos

Genome-edited bovine embryos were produced *in vitro* following protocols previously described (Lamas-Toranzo *et al.* 2019). Briefly, immature cumulus-oocyte complexes (COCs) were obtained by aspirating 2–8 mm follicles from bovine ovaries collected at local slaughterhouses. COCs were selected based on conventional morphological criteria and matured for 24 h in groups of 50 in four-well dishes containing TCM-199 medium supplemented with 10% (v/v) fetal calf serum (FCS) and 10 ng/mL epidermal growth factor (EGF) at 38.5°C and 5% CO_2_ in the air with a humidified atmosphere. Matured oocytes were denuded by vortexing for 3 min in 1 mL of 300 µg/mL hyaluronidase and randomly divided into two groups for microinjection. One group was microinjected with a solution of *Cas9* mRNA and sgRNA against *SMC2* (C+G group) at a concentration of 300 and 100 ng/µL, respectively, and another with *Cas9* alone at 300 ng/µL, serving as an injection control (C group). Cytoplasmic microinjection was performed on denuded oocytes using a filament needle under a Leica DMi8 inverted microscope assisted by Femptojet (Eppendorf).

Immediately following microinjection, IVF was carried out with frozen-thawed spermatozoa from a single stud bull selected through a gradient of 40–80% Bovipure (Nidacon Laboratories AB, Göthenborg, Sweden). Spermatozoa were co-incubated with 30–50 microinjected oocytes at a final concentration of 10^6^ spermatozoa/mL in TALP medium supplemented with 10 mg/mL heparin, in four-well plates at 38.5°C in an atmosphere of 5% CO_2_ and maximum humidity. At approximately 20 h post-insemination (hpi), presumptive zygotes were vortexed for 30 sec to remove the spermatozoa attached to the zona pellucida and cultured in groups of 20–25 in 25 μL droplets of synthetic oviduct fluid (SOF), supplemented with 5% FCS under mineral oil. Culture took place at 38.5°C in an atmosphere of 5% CO_2_, 5% O_2_, and 90% N_2_ with maximum humidity. Cleavage and blastocyst rates were evaluated at 48 hpi and at D8 post-insemination, respectively. D8 blastocysts were fixed in 4% PFA during 15 min at RT and stored in PBS-1% BSA as described above.

Post-hatching culture was carried out following an optimized protocol described in (Ramos-Ibeas *et al.* 2023). Briefly, D7 blastocysts were cultured in 500 μL of N2B27 medium (1:1 Neurobasal and DMEM/F12 medium supplemented with 1x penicillin/streptomycin, 2 mM glutamine, 1x N2 and 1x B27 supplements; Thermo Fisher Scientific). Post-hatching culture took place at 38.5°C in a water-saturated atmosphere of 5% CO_2_, 5% O_2_, and 90% N_2_ and half of the culture medium was replaced every second day. Embryos remained in culture until D12, when embryo survival was analyzed (alive embryos were able to maintain the blastocoel, whereas dead embryos collapsed). Surviving embryos were fixed as described above.

### Lineage development analysis by immunohistochemistry (IHC)

D8, D12, and E14 embryos fixed as described above, were washed in PBS-1% BSA and permeabilized in 1% Triton X-100 in PBS for 15 min at RT and blocked in 10% FCS-0.02 Tween 20 in PBS for 1 h at RT. Subsequently, specimens were incubated overnight at 4°C with primary antibodies to detect trophectoderm (CDX2, Biogenex MU392A-UC, 1:100 dilution), hypoblast (SOX17, R&D AF1924, 1:100 dilution), and epiblast (SOX2, Invitrogen 14-9811-80, 1:100 dilution) cells. After four washes in PBS-1% BSA, embryos were incubated in the appropriate secondary Alexa-conjugated antibodies (Donkey anti-rat IgG Alexa Fluor^TM^ 488, Donkey anti-goat IgG Alexa Fluor^TM^ 555, and Donkey anti-mouse IgG Alexa Fluor^TM^ 647; Life Technologies) and DAPI for 1 h at RT, followed by four washes in PBS-1% BSA. Finally, embryos were mounted on PBS-1% BSA microdrops made by drawing circles with a PAP pen (Kisker Biotech GmbH) on a coverslide (Bermejo-Alvarez *et al.* 2012). Microdrops were covered by an incubation chamber (Sigma Z359467) to prevent embryo crushing. Embryos were imaged at a structured illumination equipment composed of a Zeiss Axio Observer microscope coupled to ApoTome.2 (Zeiss). Following image acquisition, embryo diameter was measured to determine embryo size, and lineage development was analyzed. Total, CDX2+ and SOX2+ cell numbers were manually counted in D8 blastocysts using the ZEN software (Zeiss). In D12 embryos, complete hypoblast migration was determined when SOX17+ hypoblast cells expanded on the inner surface of the CDX2+ trophectoderm, and epiblast survival was identified by the presence of SOX2+ cells in the embryo, whereas ED-like formation was identified by the presence of a compact structure containing SOX2+ cells.

### Embryo genotyping

Embryo genotyping was performed following fixation and image analysis by Sanger sequencing in all E14 conceptuses collected, generated by the cross of carrier individuals. To that aim, a fragment of each conceptus was dissected and placed at the bottom of a 0.2 mL PCR tube and stored at −20 ºC until analysis. Samples were digested with 15 µL of Arcturus Picopure DNA extraction solution (Thermo Fisher Scientific) following incubation at 65°C for 1 h and inactivation at 95°C for 10 min. PCR was performed using 4 µL of the lysate in a 50 µL PCR reaction (GoTaq Flexi, Promega) to amplify the fragment containing the SNP of HH3, employing the primers described in Table 1. PCR conditions were as follows: 94°C for 2 min; ×35 (94°C for 20 s, 60°C for 30 s, 72°C for 30 s); 72°C for 5 min; hold at 8°C. The PCR products were purified using the FavorPrep^TM^ PCR Purification kit (Favorgen) and Sanger sequenced (Stabvida, Portugal) to identify non-carrier (NC, i.e. wild type, WT), single-carrier, and double-carrier embryos (Fig. 1C).

All D8 and D12 embryos generated following CRISPR-based genome editing in C+G group, were individually genotyped by Deep Sequencing (miSeq, Illumina) to identify all alleles harbored by each specimen (Lamas-Toranzo *et al.* 2019). To that aim, specimens were stored and digested as described for E14 fragments but employing a reduced amount (8 µL) of Arcturus Picopure DNA extraction solution. A first PCR was performed to amplify the sequence containing the CRISPR target site, adding Illumina adaptors using the primers detailed in Table 1 and adding 3 µL of lysate in a 25 µL PCR reaction (GoTaq Flexi, Promega). PCR conditions were as follows: 96°C for 2 min; ×32 (96°C for 20 s, 60°C for 30 s, 72°C for 30 s); 72°C for 5 min; hold at 8°C. The PCR product was purified using AMPure XP beads (Beckman Coulter^TM^) following the manufacturer´s recommendations. A second 50 µL PCR was performed with Nextera XT Index Kit v2 primers (Illumina) to add barcodes identifying each specimen, using 5 µL of each purified PCR product as a template. PCR conditions were as follows: 95°C for 3 min; ×8 (95°C for 30 s, 55°C for 30 s, 72°C for 30 s); 72°C for 5 min; hold at 8°C. The PCR products from different embryos were purified with AMPure XP beads, pooled into a 4 nM library, and sequenced at miSeq (Illumina). Reads were aligned with the WT sequence using the *BWA mem* aligner (v.0.7.17, (Li and Durbin 2009)), sorted and indexed with a pipeline of different tools from the *SAM tools* package (v.1.16.1 (Li *et al.* 2009)) and variants were called with freebayes(Garrison & Marth 2012). VCF files were processed with an in-house script to select only potential indels within the CRISPR probe editing region and passing the quality check filter. Results were confirmed visually by checking each VCF file with the Integrative Genomics Viewer (Thorvaldsdottir *et al.* 2013). Following genotyping, based on the analysis of >1000 reads of the target region/embryo, embryos were classified as wild-type (WT, containing no mutated alleles, i.e. all embryos in the C group), in-frame (IF, edited embryos containing at least one non-frame disrupting indel), and knock-out (KO, containing only alleles formed by indels non-multiple of three, i.e. frame-disrupting indels).

### Telomere length analysis

Relative telomere length was analyzed in D8 (9 embryos/genotype) and D12 (9 embryos/genotype) *in vitro* produced embryos and in all *in vivo* conceptuses obtained (6 non-carrier (NC), 9 SC, and 2 DC) by qPCR following similar protocols to those described in (Bermejo-Alvarez *et al.* 2008; de Frutos *et al.* 2016), which involve the amplification of both a telomeric sequence and another chromosomal sequence, the latter serving to normalize the number of telomere repeats to the amount of total DNA present in the sample. Telomere sequences were amplified using specific primers designed by (Callicott & Womack 2006) and the multicopy sequence Sat216 (Manna *et al.* 2003) was used to normalize the DNA amount present in the sample. Prior to the analysis, primers (shown in Table 1) were tested to display qPCR efficiencies above 0.9, and qPCR was performed by adding 2 µL of Picopure® lysate of each sample (the same employed for embryo genotyping) to the PCR mix (GoTaq qPCR Master Mix, Promega) containing specific primers. Reactions were carried out in a 20 µL volume in a MIC thermocycler (Biomolecular Systems). Fluorescence was acquired in each cycle to determine the threshold cycle. According to the comparative CT method, the ΔCT value was determined by subtracting the endogenous control Sat216 CT value of each sample from the CT value for the telomeric sequence. ΔΔCT was calculated using the highest CT group value (i.e. the group with shorter telomeres) as an arbitrary constant to be subtracted from all other sample values. Fold changes in the relative gene expression of the target were determined using the formula 2^-ΔΔCT^.

### Statistical analysis

Data were analyzed using GraphPad Prism (GraphPad Software) and SigmaStat (Systat Software, San Jose, CA, USA) packages. The chi-square test was used to analyze the differences in complete hypoblast migration, epiblast survival, and ED-like formation rates between groups. Differences in blastocyst rates between groups C and C+G were analyzed by *t*-test using the blastocyst rate of each experimental replicate (i.e. % of zygotes reaching the blastocyst stage for each group in each replicate) as input data. Differences in D7 to D12 embryo survival (input data being similar to those for blastocyst rate analysis), embryo diameter, and cell numbers between different genotypes (3: WT, IF, and KO) were analyzed by one-way ANOVA. When the normality test failed, statistical differences were analyzed by non-parametric *t*-test (Mann–Whitney) or one-way ANOVA (Kruskal–Wallis test).

## Results

### HH3 DC conceptuses fail to develop to E14

A total of 17 structures were recovered at E14 from 4 superovulated HH3 carrier cows inseminated with a carrier bull. Genotyping revealed that 2/17 were DC (Table 2), which did not deviate significantly (*P* > 0.05) from the expected 1/4 Mendelian proportion. Nevertheless, HH3 DC structures showed severe developmental defects. In contrast to their NC (WT) and SC counterparts, which were tubular or filamentous, DC structures remained spherical and were significantly smaller (<0.5 mm) than their NC and SC counterparts, evidencing a severe defect in the proliferation of extra-embryonic membranes (Fig. 1B, Table 3). Immunohistochemistry detected embryonic discs formed by SOX2+ epiblast cells in all 6 NC and 9 SC conceptuses analyzed, but no epiblast cells (the lineage from which the embryo proper derives) could be detected in the 2 DC structures. The differentiation of the hypoblast (an extra-embryonic lineage derived from the inner cell mass) was not affected in HH3 DC structures, as complete hypoblast migration was observed in all structures analyzed irrespective of their genotype.

**Table 2:** Number of non-carrier (NC), single-carrier (SC), and double-carrier (DC) conceptuses obtained from 4 crosses between HH3 SC individuals. Mendelian proportion was observed (chi-square test, *P* > 0.05).

	Cow 1	Cow 2	Cow 3	Cow 4	Total
Conceptuses recovered	7	3	3	4	17
NC conceptuses	1	1	1	3	6 (35.3%)
SC conceptuses	5	1	2	1	9 (52.9%)
DC conceptuses	1	1	0	0	2 (11.8%)

**Table 3:** Conceptus elongation and lineage development in non-carrier (NC), single-carrier (SC), and double-carrier (DC) conceptuses. Different letters in the same column indicate statistically significant differences (ANOVA and chi-square test, *P* < 0.05).

	NC	HH3 SC	HH3 DC
Conceptus length (mm; mean ± s.e.m.)	14.1 ± 4.1^a^	5.6 ± 2.6^ab^	0.4 ± 0.0^b^
Complete hypoblast migration	6/6	9/9	2/2
Embryonic disc formation	6/6	9/9	0/2

### *SMC2* KO embryos show reduced cell proliferation at D8 and fail to develop to D12 *in vitro*

To determine when the developmental defects of HH3 DC arise, *SMC2* KO embryos were generated *in vitro*. To that aim, a group of oocytes was microinjected with Cas9 mRNA and sgRNA targeting the region of the *SMC2* gene affected by the HH3 mutation (group C+G, partially composed of *SMC2* KO embryos) and another with Cas9 mRNA (group C, an injection control only composed of WT embryos). Developmental rates (cleavage at 48 hpi and blastocyst rate at D8) were similar between C+G and C groups, suggesting that *SMC2* disruption did not impair embryo development to the blastocyst stage (Table 4).

**Table 4:** Developmental rates in C and C+G groups up to the blastocyst stage. The number of replicates and microinjected oocytes in all *in vitro* experiments, as well as cleavage and blastocyst rates in control (C) and experimental (C+G) groups, are presented (*t*-test, *P* > 0.05).

	C group	C+G group
*n*	4	4
Microinjected oocytes	192	304
Cleavage rate	161/192	240/304
Mean ± s.e.m.	83.4 ± 1.7	78.9 ± 0.8
Blastocyst rate	56/192	83/304
Mean ± s.e.m.	27.0 ± 4.5	26.6 ± 3.4

**Figure 2:**
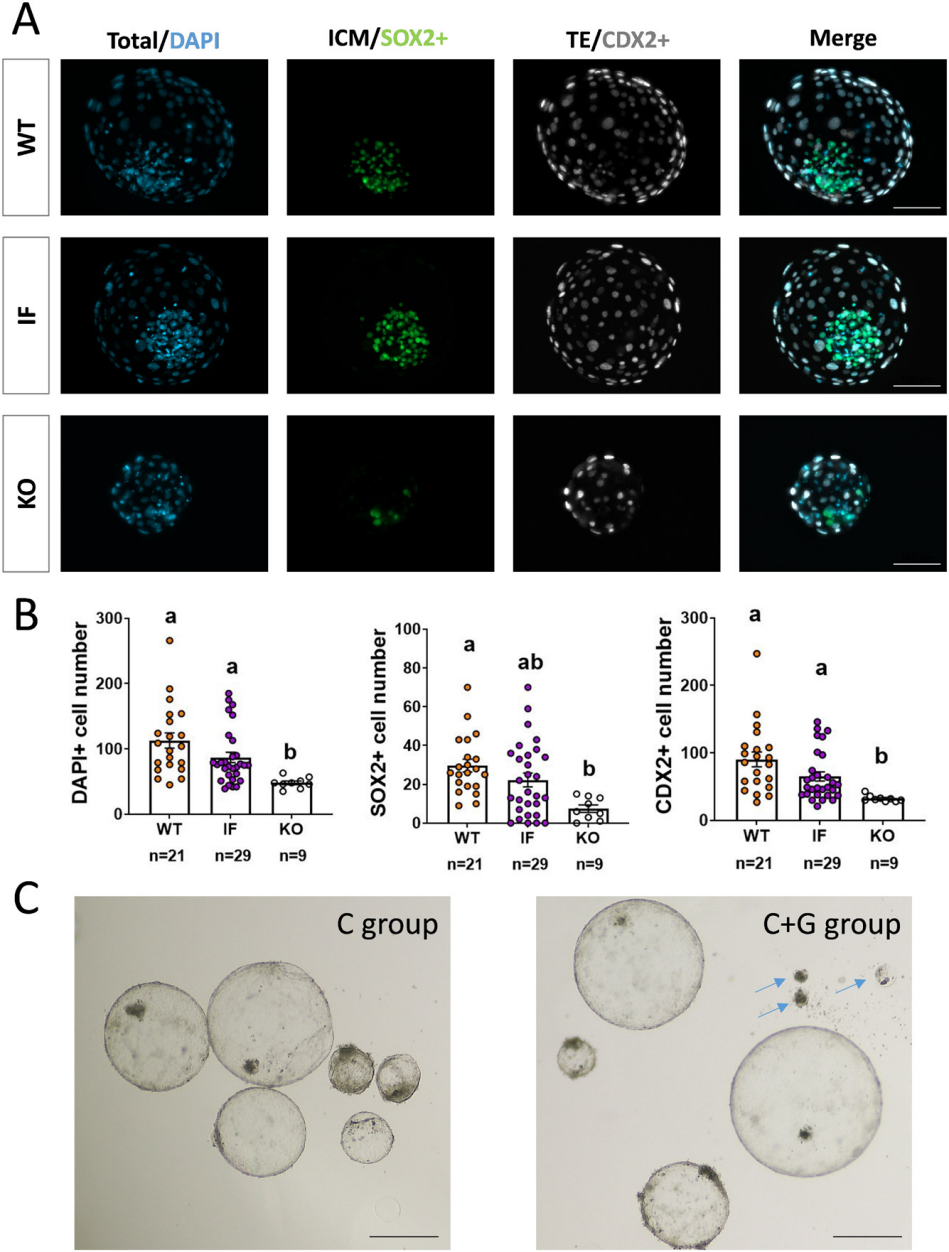
Development of cell lineages in D8 blastocysts. A) Representative pictures of WT, edited in-frame (IF), and *SCM2* KO blastocysts after immunohistochemistry for SOX2 (ICM) and CDX2 (TE); nuclei counterstained with DAPI. Scale bar: 100 µm. B) Scatter plots of DAPI+, SOX2+, and CDX2+ cell numbers in WT, IF and *SCM2* KO blastocysts. Different letters indicate statistically significant differences between groups (mean ± s.e.m.; Kruskal–Wallis test, *P* < 0.05). C) Representative pictures of D12 structures in C and C+G groups. Dead structures (KO embryos) showing a collapsed blastocoel are indicated with arrows. Scale bar: 500 µm.

Genotyping of 38 blastocysts from C+G group revealed the presence of 9 KO embryos (which harbor only KO alleles composed of frame-disrupting indels) and 29 edited IF embryos (containing at least one IF allele), resulting in a 100% edition efficiency and ~24% KO generation efficiency, comparable to previous reports (Perez-Gomez *et al.* 2024*a*, *b*). *SMC2* KO blastocysts were formed by a significantly lower number of total, trophectoderm (CDX2+), and inner cell mass (SOX2+) cells compared to their WT counterparts (Fig. 2A and B), evidencing that *SMC2* ablation impaired cell proliferation during early embryo development.

To evaluate the developmental potential of *SMC2* KO embryos beyond the D8 blastocyst stage, a post-hatching *in vitro* culture system was employed to assess embryo development up to D12 (Ramos-Ibeas *et al.* 2020). To determine embryo survival rates for each genotype, both alive and dead structures – the latter identified as collapsed structures unable to maintain their blastocoel (Fig. 2C) – were genotyped. Genotyping revealed that none of the 9 D7 *SMC2* KO blastocysts were able to survive to D12, whereas most (14/19) WT embryos developed to D12 (Table 5).

**Table 5:** Survival rates from D7 to D12 of wild type (WT), edited in-frame (IF), and knock-out (KO) embryos. Number of D12 surviving embryos following post-hatching *in vitro* culture. Different letters in the same column indicate statistically significant differences (chi-square test, *P* < 0.05).

Group/genotype	*n*	Survival rate (%)
C		
WT	2	14/19 (73.7)^a^
C+G	2	
IF		15/32 (46.9)^a^
KO		0/9 (0)^b^

### Relative telomere length was not altered in embryos lacking condensins

**Figure 3:**
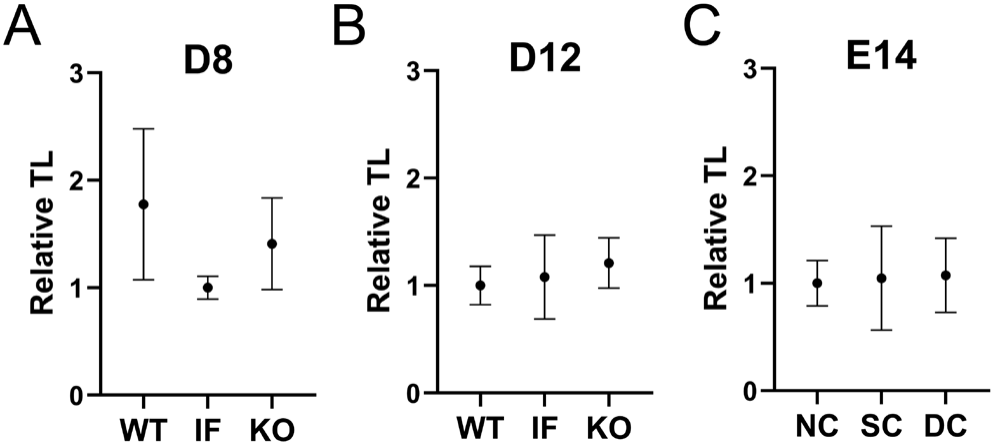
Relative telomere length in bovine embryos lacking condensins I and II. A) Relative telomere length in WT, IF, or *SMC2* KO D8 blastocysts. B) Relative telomere length in WT, IF, or *SMC2* KO D12 structures. C) Relative telomere length in NC, SC, or DC E14 conceptuses for HH3 allele. No significant differences were observed between groups (mean ± s.e.m.; Kruskal–Wallis test, *P* > 0.05).

Relative telomere length was similar in *SMC2* KO D8 blastocysts compared to IF or WT counterparts (Fig. 3A). No differences were observed either in relative telomere length between *SMC2* KO, IF, or WT D12 structures and between non-carrier, single-carrier, or double-carrier E14 conceptuses for HH3 haplotype (Fig. 3B and C).

## Discussion

Condensins I and II are large protein complexes involved in processes requiring large-scale rearrangements of chromatin structure, including mitosis (reviewed by (Hoencamp & Rowland 2023)). Both condensins share the SMC2–SMC4 heterodimer, and therefore the ablation of any of these proteins prevents the formation of both complexes (Hirano & Mitchison 1994). Although substantial experimentation on the role of condensins has been conducted in cell cultures (e.g. Hirano & Mitchison 1994; Ono *et al.* 2003; 2004), the developmental ability of mammalian embryos lacking condensins has not been investigated, as the only report for *Smc2* ablation in mice was not focused on embryo development but on neural stem cells (Nishide & Hirano 2014), and no data on *Smc4* ablation in any mammalian embryo is publicly available. Our results show that the developmental effects of *SMC2* ablation in bovine embryos are noticeable before blastocyst hatching, as reduced cell proliferation was observed in KO embryos by D8, highlighting the involvement of condensins I and II in blastomere mitosis. However, *SMC2* KO embryos were able to undergo first lineage differentiation and form a functional trophectoderm able to generate a blastocoel by D8, although embryo development was arrested soon after blastocyst hatching. This observation coincides with the phenotype of naturally occurring HH3 DC embryos, which were unable to undergo elongation and had lost the lineage that would eventually form the fetus (the epiblast) before gastrulation. Although the number of HH3 DC embryos analyzed is limited due to the inherent difficulties of obtaining embryos from carrier animals under the milk production cycle, the clear developmental arrest observed at earlier stages (D12 roughly equivalent to E10 (Ramos-Ibeas *et al.* 2020)) confirms the early developmental arrest of embryos lacking condensins.

The combination of CRISPR technology and conventional (D0–D8) and advanced (D7–D12) *in vitro* embryo culture systems (Ramos-Ibeas *et al.* 2023) constitutes a powerful tool to test the developmental effects of any mutation, surpassing some of the limitations inherent to experimentation in large animals. However, both technologies have limitations. In the case of conventional CRISPR/Cas9 technology, KO generation rates are often below 40% despite the significantly higher edition rates (100% for the gdRNA used herein) due to the frequent presence of in-frame alleles formed by indels multiples of three (Lamas-Toranzo *et al.* 2019). The effect of in-frame indels on protein functionality is largely unpredictable, and therefore they cannot be deemed KO alleles, which necessitates careful genotyping techniques able to identify all the alleles harbored by an edited embryo. Cytosine base editors (Komor *et al.* 2016) constitute a plausible alternative to the conventional CRISPR/Cas9 system, as they can introduce a stop codon with very high efficiency, and the substitution of single bases instead of their deletion or insertion greatly facilitates greatly genotyping (Perez-Gomez *et al.* 2024*b*). Regarding the post-hatching *in vitro* culture system, it allows the assessment of hypoblast differentiation and migration (i.e. yolk sac formation), the initial growth of extra-embryonic membranes, and the formation of an early embryonic disc. Unfortunately, critical landmarks of embryo development beyond ~E10, including embryonic disc growth, complete gastrulation, and massive proliferation of extra-embryonic membranes, cannot be achieved for cattle embryos under the currently available systems, although significant advances in epiblast development have been achieved in other ungulates (Ramos-Ibeas *et al.* 2022). This has not been a limitation for this study, as the developmental effect was observed before ~E10, but it draws a clear frontier on the developmental processes that can be studied in cattle embryos under a fully *in vitro* system.

The accurate identification of the timing of developmental arrest of HH3 DC embryos is economically relevant for reproductive management in dairy farms, where the inadvertent cross between heterozygous individuals (single-carriers, SC) results in a ~25% probability of generating an unviable DC embryo. Unfortunately, the carrier status of most heifers worldwide is still unknown and carrier bulls are still in use, resulting in a significant number of crosses between carrier individuals. The economic loss associated with embryonic or fetal death varies greatly depending on when DC embryos arrest their development. Pregnancy losses occurring beyond maternal recognition of pregnancy delay luteolysis, increasing both the non-productive periods (open days) and the risk of uterine pathologies associated with abortions if the DC embryo achieves implantation (Wiltbank *et al.* 2016). Fortunately, HH3 DC embryos arrest their development prior to maternal recognition of pregnancy, which is triggered by the secretion of interferon Tau (IFNT) from the conceptus (Helmer *et al.* 1989; Imakawa *et al.* 1987). HH3 DC conceptuses did not reach the minimum 5 mm size limit required to produce enough IFNT to prevent luteolysis (Mann & Lamming 2001). In this perspective, the embryonic losses associated with the HH3 haplotype are roughly equivalent to non-fertilizing insemination, as – in the absence of signals preventing luteolysis – the estrous cycle will resume timely, causing a minimal increase in open days.

Beyond the interest in cattle reproductive management, these results prove that condensins are essential to sustain mitotic divisions during early mammalian embryo development, although KO embryos were able to undergo ~6 cell divisions in 8 days (~50 cells). Given the essential role of condensins during mitosis, KO embryos (unable to produce SMC2 *de novo*) may have been able to undergo several cell divisions due to the retention of oocyte-stored SMC2 protein. At the early blastocyst stage, both lineages (ICM and TE) showed reduced proliferation, but only TE cells were detected at later stages. A plausible explanation is that the aneuploidy induced by condensin ablation could be better sustained by TE cells than epiblast cells. This hypothesis is supported by previous findings in human embryos that observed that aneuploid cells are specifically excluded from ICM-derivatives at post-hatching stages (Starostik *et al.* 2020) and that aneuploid stem cells can only form TE cells in human gastruloid models (Yang *et al.* 2021). As DC or KO embryos arrest their development due to failures in chromosome assembly and segregation during mitosis – well documented in cells lacking condensins (Hirano 2016) – we explored if those failures were associated with changes in telomere length, a parameter that has been associated with genome stability in cell lines (Murnane 2010; Zhao *et al.* 2023) and chromosome abnormalities in human embryos (Mania *et al.* 2014; Treff *et al.* 2011). We found no significant differences in telomere length between embryos lacking condensins (KO or DC) and WT embryos, which suggests that telomere shortening may be a cause (not a mere consequence) of the chromosomal abnormalities observed in human embryos displaying short telomeres (Mania *et al.* 2014; Treff *et al.* 2011).

In conclusion, condensins I and II are required for blastomere mitosis during early development. Bovine embryos lacking *SMC2* – a core component of condensins I and II – are able to develop to the blastocyst stage, but they show reduced cell proliferation and arrest their development shortly after blastocyst hatching, failing to elongate and trigger maternal recognition of pregnancy.

## Declaration of interest

The authors declare that there is no conflict of interest that could be perceived as prejudicing the impartiality of the study reported.

## Funding

This work was supported by the projects StG-757886-ELONGAN from the European Research Councilhttp://dx.doi.org/10.13039/501100000781 and PID2020-11750RB-I00 and ECQ2018-005184-P from the Spanish Ministry of Science and Innovation to PBA.

## Author contribution statement

APG, IFB, BGC, and JGH conducted in vitro embryo production experiments. APG, IFB, and BGC performed embryo genotyping and IHC analyses. APG and ILT conducted oocyte microinjection, and LGB provided in silico analyses. IFB conducted relative telomere length analysis. PRI and PBA supervised and designed experiments and managed funds. APG and PBA wrote the manuscript, which was revised by all authors.
